# Stability Calculation Method of Slope Reinforced by Prestressed Anchor in Process of Excavation

**DOI:** 10.1155/2014/194793

**Published:** 2014-02-11

**Authors:** Zhong Li, Jia Wei, Jun Yang

**Affiliations:** ^1^Key Laboratory of Disaster Prevention and Mitigation in Civil Engineering of Gansu Province, Lanzhou University of Technology, Lanzhou 730050, China; ^2^Western Engineering Research Center of Disaster Mitigation in Civil Engineering of Ministry of Education, Lanzhou University of Technology, Lanzhou 730050, China

## Abstract

This paper takes the effect of supporting structure and anchor on the slope stability of the excavation process into consideration; the stability calculation model is presented for the slope reinforced by prestressed anchor and grillage beam, and the dynamic search model of the critical slip surface also is put forward. The calculation model of the optimal stability solution of each anchor tension of the whole process is also given out, through which the real-time analysis and checking of slope stability in the process of excavation can be realized. The calculation examples indicate that the slope stability is changed with the dynamic change of the design parameters of anchor and grillage beam. So it is relatively more accurate and reasonable by using dynamic search model to determine the critical slip surface of the slope reinforced by prestressed anchor and grillage beam. Through the relationships of each anchor layout and the slope height of various stages of excavation, and the optimal stability solution of prestressed bolt tension design value in various excavation stages can be obtained. The arrangement of its prestressed anchor force reflects that the layout of the lower part of bolt and the calculation of slope reinforcement is in line with the actual. These indicate that the method is reasonable and practical.

## 1. Introduction

In the structure of prestressed anchor frame beam, the reinforcement mechanism of the combination of anchor and frame beam is through the powerful prestress to strengthen prereinforcement slope. When the slope is under the action of prestress, first, physical, and mechanical performance of the rock and soil body under the action of the prestress can be improved to a certain extent, and it can make full use of the capacity of rock and soil body and enhance the stability of slope. Secondly, pressure of the sliding body vertical to the sliding surface is increased obliviously, so sliding friction force on the surface of the slip is increased, and thereby the stability of slope body is improved. Frame ground beam mainly can pass the anchoring force; at the same time, it can strengthen the efficiency of the structure.

As for the research of prestress anchor frame beam, it is mainly focused on prestress anchor mechanism, calculation method of frame beam internal force, and some field test research, but all these mainly aim at the internal force calculation of frame beam [1–5]. From the function of prestress anchor grillage beam, the stability of reinforced slope should be the core of calculation, whether the frame structure design and calculation are reasonable should be judged according to the stability of the slope after the reinforcement of it. If the reinforcement design and slope stability are relatively independent of each other, on the one hand, it can lead to the instability of slope, and on the other hand, it will cause unnecessary waste.

As to these problems, the simulation research is conducted through the related software, but quantitative judgment and design for the slope stability cannot be done, especially for the new type of anchorage structure which is similar to prestress anchor frame beam [6, 7].

Reinforcing slope presents great changes compared with traditional slope, the stability analysis of slope stress system is relatively more complex, and the related research is rare. The author has made some research on the problem [8], but this method does not take the relationship between bolt arrangement and excavation height in slope excavation process into consideration, and the analysis of slope stability in excavation of different stages in the process cannot be made in real time.

When the stability of slope is calculated by using the limit equilibrium theory, the most prominent problem is the failure mode of slope and the most dangerous slip surface of slope position; in general, this problem is no longer difficult. However, as to the new prestressed anchor frame beam reinforcement structure, when performing analysis of the stability of the slope, the stability analysis is more complex. At this time, the effect of additional function of retaining structure on the slope stability becomes the main problem, and the general slope stability problems have the same points, and also highlight the characteristics of their own. Thus, the soil structure interaction, anchor, under the condition of prestressed anchor lattice framework should be considered in the establishment of calculation model of reinforced slope stability, and the stability calculation also should be taken as the core. In the study, the analysis method of different stage of excavation slope in the critical state of tension and the analysis of program design and examples are given out.

## 2. Reinforcement Mechanism of Prestressed Anchor Framework

**Figure 1:**
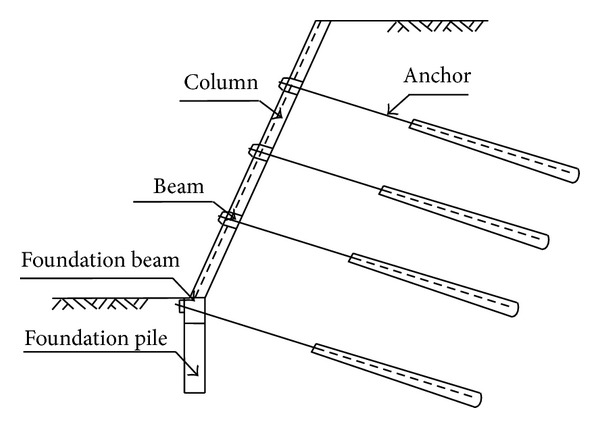
Sectional sketch of grillage supporting structure with prestressed anchor.

The framework beam is the prestressed anchor frame beam structure; it consisted of anchor and soil body, and it belongs to the light soil retaining structure. Frame part forms beam frame structure system, the head of anchor rod connected with the frame in the intersection of the column and beams, inner end anchor is fixed in the soil, the soil of frame beam is transmitted from anchor head to steel rod, and then, through the friction, it is passed to the surrounding soil of anchorage zone to support the earth pressure force of frame structure. In the process of work, the structure can strengthen the anchor beam of frame and guarantee bolt uniformity in the resistance to sliding, continuity, and integrity and achieve the goal of completely stable slope. Prestressed anchor frame beam supporting structure model is shown in Figure 1.

## 3. Stability Calculation Model of Frame Supporting Structure with Prestressed Anchor

**Figure 2:**
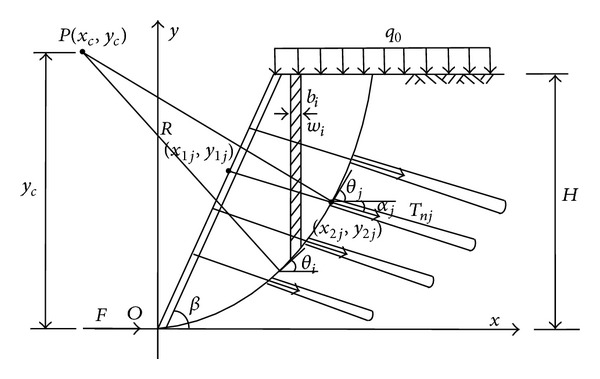
Stability analysis of grillage supporting structure with prestressed anchor.

Based on the failure mode of soil slope, it takes the soil, frame structure, and anchor rod (rope) interaction into consideration, and, according to the literature, for soil slopes and large-scale rock slope structure circular sliding method is appropriate. Stability calculation model of prestressed anchor frame beam is shown in Figure 2.

Point *O* is taken as the slope foot, and we establish a coordinate system; *P*(*x*_*c*_, *y*_*c*_) represents the circular slip surface center of the circle, (*x*_1*j*_, *y*_1*j*_) represents the *j* layer coordinate of anchor arm point, (*x*_2*j*_, *y*_2*j*_) represents the *j* layer intersection coordinate of anchor point and glide plane, *H* is the height of slope; the gear structure and slip plane within the soil are taken as a whole, anchor in the slip surface is called freedom segment, and outside slip surface is called anchorage segment.

The main force of slope suffered is its gravitation, the tension *T*_*nj*_ along the direction of anchor effect of anchor rod, and the horizontal thrust *F* at the bottom of it.

From the stability coefficient, the solution of the most dangerous slip surface and the layers of anchor rod tension can be calculated. The calculation method involves three aspects; prestressed anchor framework reinforcement stability is put forward below.

### 3.1. Solving Stability Coefficient

As shown in Figure 2, through the limitation equilibrium theory, as to certain given slip plane position, the influence of the frame beam and anchor on slope stability should be taken into consideration; the bishop method is adopted in improving the stability, and the coefficient calculation types are as follows:

(1) $$\begin{array}{c} F_{s}=\frac{M_{R}}{M_{T}}, \end{array}$$

(2) $$\begin{array}{c} M_{R}=M_{s}+M_{a}+F\cdot y_{0}, \end{array}$$

(3) $$\begin{array}{c} M_{s}=\sum_{i=1}^{n}\frac{1}{m_{\theta i}}\left[c_{i}L_{i}+\left(w_{i}+q_{0}b_{i}\right)\cos\theta_{i}\cdot tg\varphi_{i}\right]\cdot s\cdot R, \end{array}$$

(4) $$\begin{array}{c} m_{\theta i}=\cos\theta_{i}+\frac{tg\varphi_{i}}{F_{s}}\sin\theta_{i}, \end{array}$$

(5) $$\begin{array}{c} M_{a}=\sum_{j=1}^{m}T_{nj}\times \left[\cos\left(\alpha_{j}+\theta_{j}\right)+\sin\left(\alpha_{j}+\theta_{j}\right)\cdot tg\varphi_{jk}\right]\cdot R, \end{array}$$

(6) $$\begin{array}{c} M_{T}=\left[s\gamma_{0}\sum_{i=1}^{n}\left(w_{i}+q_{0}\cdot b_{i}\right)\sin\theta_{i}\right]\cdot R. \end{array}$$

Among them, *F*_*s*_ represents the safety factor; *M*_*R*_ represents the total resistance torque of the sliding surface; *M*_*T*_ represents total declining torque on sliding surface; *M*_*s*_ represents the antisliding torque of soil and the upper load; *M*_*a*_ represents the equivalent resistance torque generated by tensile resistance; *F* represents design values of the horizontal thrust at the bottom of frame structure; *y*_0_ represents the distance from circle center of arc slip surface to the bottom of frame structure. *n* represents the slip number of sliding body; *m* represents the layer number of anchor; *γ*_*k*_ represents coefficient of integral sliding subentry; *γ*_0_ represents the importance coefficient; *ω*_*i*_ represents the soil weight of *i* layer; *b*_*i*_ represents the width of *i* layer; *c*_*ik*_ represents standard values of cohesive strength on critical slip surface; *φ*_*ik*_ represents standard value of internal friction angle on the sliding surface; *θ*_*i*_ represents the angle between the tangent line and the horizontal plane at any point on the critical slip surface. *α*_*j*_ represents the angle between anchor rod and the horizontal plane; *L*_*i*_ represents the arc length of the slip surface; *s* represents the thickness of sliding body unit; *R* represents radius of slip plane arc; *F* represents design value of horizontal thrust at the bottom of the frame beam; *T*_*nj*_ represents the tensile resistance of the *j* layer anchor arm.

### 3.2. Search Model of Potential Sliding Surface

According to the sliding surface basic characteristics such as shape, size, and location when the soil slope slip occurs, it puts forward two assumptions as qualification of sliding surface search, based on these assumptions, the search of sliding surface can be realize, and the actual situation of the slip plane can be obtained.

**Figure 3:**
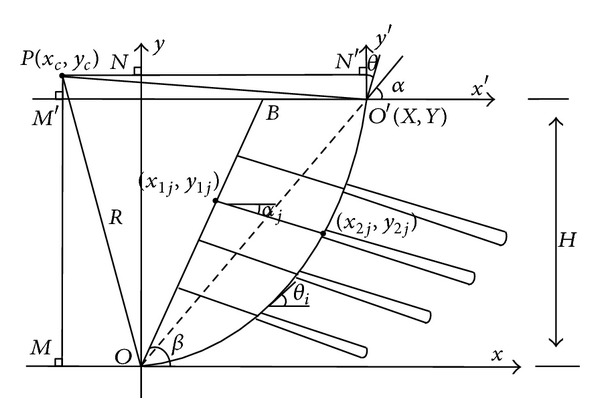
Slip search model of slope reinforced by prestressed anchor.

1. Two assumptions.
  - If we assume that under the effect of slope retaining structure, the slip plane is passed through the slope foot points *O*, which is shown in Figure 3.
  - The slope surface and slip plane tangent arc intersection point *O*′ should have horizontal angle, which is not greater than 90°.
2. The sliding surface search model.

The following model of prestressed anchor frame beam and sliding surface search way is the same as the model proposed in paper [6], which is shown in Figure 3; the lines $\bar{OB}$, $\bar{BC}$ represent the slope surface, points *O* represent slope bottom point, circular arc *OO*′ is the slope slip plane point pass through the *O* and it intersects with slope surface in point *O*′, and the point *P* is the center of slip plane circle.

Take the *O* and *O*′ as the origin point of two rectangular coordinate system; the coordinate system with the origin *O* is taken as the basic coordinate system, as coordinate system with the origin *O*′ is taken as auxiliary coordinate system; lines *PN* and *PM* are projection in the *x* direction and *y* direction, respectively, in the basic coordinate system. The lines *PN*′ and *PM*′ are the projection in the *x*′ and *y*′ direction of the auxiliary coordinate system.

The points (*x*_1*j*_, *y*_1*j*_)(*x*_2*j*_, *y*_2*j*_) represent intersection points of the first layer and the slope surface and slip plane circular; (*x*_1*j*_, *y*_1*j*_) can be calculated by the design parameters; (*x*_2*j*_, *y*_2*j*_) can be obtained through the anchor in the equation of the straight line and arc equation. In Figure 3, *X*, *Y*, *θ* represent the horizontal angle (*X*, *Y*) and arc *O*′; the three variables are both related to the *O*′ point. The derived process of sliding surface search model is as described as in the paper [6]. The function of variables is shown in the following type

(7) xc=tan⁡θ·(X2+Y2)2(Y−tan⁡θ·X)+X,yc=X2+Y22(tan⁡θ·X−Y)+Y.

The slope model is shown in Figure 3, when the sliding surface is searching, each search variable changes according to the following formula

(8) Xi=H·cot⁡β+i·ΔX, i=1,2…n,Yi=H, Xi∈BC−,θj=αi+j·Δθ, j=1,2…n,αi=arctan⁡(YiXi), i=1,2…n,

where *β* represents the angle between slope surface and horizontal plane, *H* is the slope height, and *α*_*i*_ is the angle between the slip plane arc $\bar{OO^{\prime }}$ and horizontal plane.

### 3.3. Stability of the Excavation Process Based on Each Layer Anchor Tension

In the supporting design of prestressed anchor framework, the stability is the core content of the design; the design shall be based on stability of the supporting structure and take it as the prerequisite. Therefore, in the stability analysis, the first step is calculation of the anchor tension and design of anchor and then the system design of the bearing capacity of frame.

With the change of retaining wall, anchor rod anchor force, and the structure effect, safety coefficient will be changed accordingly. The stability analysis of prestressed anchor frame beam in the process of the excavation will increase a lot uncertainty of stability, it is not conformity with the actual situation according the value of safety coefficient to determine whether the slope is stable or not.

Aiming at these problems, on the basis of safety coefficient, certain constraint about the relationship between the position and force of each layer anchor can be given in all stages of the slope excavation, ultimately the theoretical calculation method and a optimal solution can be obtained.

(1) The design of the layers of anchor rod tension must meet the requirements of stability, namely, that

(9) $$\begin{array}{c} F_{s}-1.3\geq 0. \end{array}$$

The expression and solution of *F*_*s*_ can be obtained from (1)–(6).

(2) According to the construction process of prestressed anchor frame beam and slope position and slope excavation at various stages, the relationship between the heights of each layer in the various stages of slope excavation can be established; the unification of the anchor tension calculation formula of the specific derivation process is shown below.

**Figure 4:**
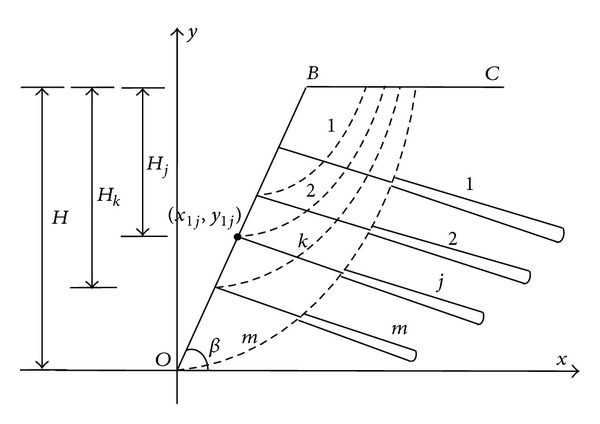
Anchor arrangement and slope excavation stages.

As shown in Figure 4, from top to bottom of slope, the total layer number is set *m*; when construction of slope excavation is started, according to the arrangement of each layer anchor, it can be divided into many stages of excavation.

In addition, according to the stability of prestressed anchor frame beams as shown in Figure 2, when excavation is performed from top to bottom of slope, the larger the deeper, the greater the slope stability. The layers of the size of the anchor tension are related to its layout, as, for the high slope, the middle and lower parts of slope is the key to slope reinforcement, usually where the bolt is under pulling force. Therefore, in order to make analysis of the influence of anchor arrangement on anchor rod tension, the following types are presented:

(10) $$\begin{array}{c} \alpha_{jk}=\{\begin{array}{cc} \frac{2H_{j}}{H_{k}} & H_{j}\leq \frac{H_{k}}{2}\\ \frac{H_{j}}{H_{k}} & H_{j}>\frac{H_{k}}{2}, \end{array} \end{array}$$

(11) $$\begin{array}{c} T_{njk}=\alpha_{jk}T_{nk}, \end{array}$$

where *α*_*jk*_ is the anchor tension influence coefficient, 0 < *α*_*jk*_ < 1, *H*_*j*_ represents the distance from the anchor end to the slope top in the layer *j*. *H*_*k*_ represents the height of slope excavation of the slope of number *k*. *α*_*jk*_ represents the influence coefficient. *T*_*njk*_ represents the tension values in the excavation in the *k* layer; *T*_*nk*_ is tension value in the stage *k* of excavation. The coordinate system as the following is proposed, in the further solving of the equation, and (*x*_1*j*_, *y*_1*j*_) represents the coordinate values:

(12) Hj=H−y1j,Hk=H−y1(j+1),

where *H* represents the slope height, *y*_1*j*_, *y*_1(*j*+1)_, respectively represent the corresponding coordinates of *j* and *j* + 1 layer anchor.

According to the above-derived formulas (10), (11), and (12), when the layout of each layer anchor coordinates is certain, given initial value, each layer anchor tension is substituted into type (5), through related calculation, anchor rod tension values meet with the stability of the slope excavation can be gotten.

(3) Solving tension value which satisfies the stability of anchor rod in each excavation stage.

According to type (11), each layer anchor tension in each excavation stage can be expressed as the following:

(13) $$\begin{array}{c} T_{nJK}=\alpha_{JK}\cdot T_{n}^{\prime }, \end{array}$$

where

(14) Tn′={Tn1    Tn2    Tnk    ⋯    Tnm},αJK=[α11          α12α22        α13α23αjk      …          α1mα2mαjm……αmm],TnJK=[Tn11          Tn12Tn22        Tn1kTn2kTnjk      …          Tn1mTn2m…Tnjm…Tnmm];

*T*_*n*_ represents the pulling force in each excavation stage located in the bottom anchor arm; *α*_*JK*_ is the influence coefficient matrix of anchor tension; *T*_*nJK*_ is the anchor tension matrix of each layer in each excavation stage.

Thus, a set of solution which can satisfy the stability of each excavation stages at the same time can be obtained by taking the maximum value of each layer anchor tension to form a new row vector according to the layer number of the anchor. After the calculation of excavation stability in each stage, the layers of anchor rod tension can be determined; it can be represented by the following type:

(15) $$\begin{array}{c} T_{nj}=\max\left\{\begin{array}{cccc} T_{nj1} & T_{nj2} & \ldots & T_{njm} \end{array}\right\}, \end{array}$$

where *j* = 1,2,…, *m* represents the layer number of anchor arm.

## 4. Calculations and Analysis

According to the calculation method of the prestressed anchor frame beam supporting structure stability design and related calculation program, in order to verify the rationality of the calculation method described in this paper, calculation and analysis are conducted below.

### 4.1. Introduction of Project

**Figure 5:**
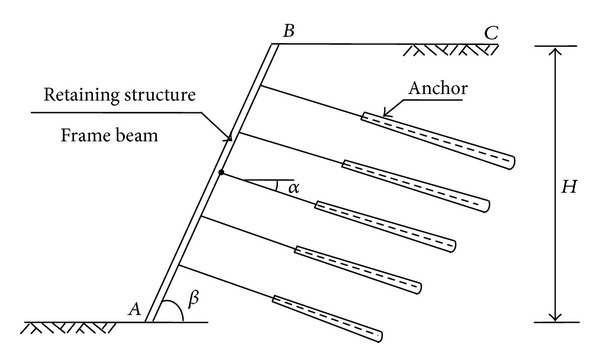
Scheme of slope reinforcement.

In some city residential areas, it needs to do amplification of land construction area as the need of project. It tends to do the slope excavation and make the related reinforcement, in order to keep good stability. Support scheme of the slope is shown in Figure 5, because the slope vertical height is *H* = 12 m and the angle between the excavation slope line and the ground is *β* = 80°. Slope with prestressed anchor frame beam supporting structure reinforcement is adopted. Along the slope height 5 layer anchors are set along the height of space layout, level of anchors spacing is about 2.5 m, the angle between layers of anchor rod and horizontal plane is 10°, uniformly distributed load on the slope surface is *q*_0_ = 10 kN/m^2^, and slope parameters are shown in Table 1.

### 4.2. Stability Analysis and Calculation

#### 4.2.1. The Slope Reinforcement Initial Parameter Calculation Method

Supporting scheme is shown in Figure 5, arrangement of each layer anchor and the height of each excavation stage can be adopted, and the initial design parameters are shown in Table 2.

#### 4.2.2. Stability Calculation and Analysis of Excavation Stage

Input the initial value to the compiled calculation program; the main calculation process is as follows: first through the method of dichotomy search values of *T*_*n*_′ in every excavation segment, and the excavation stage can be obtained through type (13), then put each layer anchor tension value into stability calculation formulas (1)–(6); through the computer, the most dangerous sliding surface search can be automatically determined, and corresponding safety factor is obtained, when the safety factor is greater than the allowed value; the safety coefficient calculation for the next excavation stage is started. Repeat such operations; the requirements of the tension matrix *T*_*nJK*_ can be obtained, and the excavation stages is shown in Table 3.

It can be seen in Table 3 that, in each layer anchor tension in each excavation stage can be maximized; at the same time, the stability of the optimal solution at various stages is obtained.

It shows that the optimal solution should have certain security reserve; from the anchor tension size along the slope height, the distribution of the middle and lower parts of slope anchor tension is bigger, and it is in accordance with the actual slope situation.

Thus, when tension value of each layer anchor is taken as maximum in each excavation, the tension optimal solution of the excavation stages can be obtained at the same time in each layer anchor. In the process of stability calculation, the variable's value and its center coordinates and radius of most dangerous sliding surface search are shown in Table 4.

As shown in Table 4, the slope slip plane of the circle coordinates and radius, in each stage of excavation, sliding on the slope surface point location is given out.

Compared with the working situation *A* and *B*, it can be found that the slope position of the most dangerous sliding surface changes with each layer anchor tension values. These phenomenon are completely different from the situation without considering the effect of retaining structure. As a result, it shows that in the calculation of the stability of the supporting structure under the action of the slope excavation process, it has obvious superiority by using dynamic search model to determine the most dangerous sliding surface potential slip surface.

## 5. Conclusion

In the process of stability analysis of prestressed anchor frame, taking the influence of supporting structure anchor on the soil slope stability into consideration, stability control is proposed based on the excavation process of prestressed anchor framework reinforcement method; the main conclusions are obtained as follows.

Considering the influence of the supporting structure and anchor on the slope stability, the most dangerous slip plane of slope changes with the design parameters are dynamic as shown in Table 4. The dynamic search model of determining the most dangerous sliding surface has the obvious superiority; it can get more accurate and reasonable result compared with stability calculation of the most dangerous slip plane ignoring the structure under the action of retaining wall and it has universality and practicability.

According to the supporting stability control characteristics of the construction process of prestressed frame anchor and relationships between the layers of anchor arrangement at various stages and the height of slope excavation, the unified calculation formula of excavation is established, and the optimal solution which is satisfied with each excavation stage on the stability is obtained.

Through calculation, from the distribution anchor tension along the slope height, in the middle and lower parts of slope anchor tension is larger; it is consistent with the fact that the slope supporting usually focuses on the middle and lower parts of slope at the time of the slope reinforcement. It proved that the rationality and practicability of the presented method in the paper.

Calculation model proposed in the paper has certain assumption in the shape of slip surface, and so forth. For some complex geological engineering of slope reinforcement, the applicability of the method is not enough. Therefore, it has important scientific and practical significance how to deeply consider the interaction between the supporting structure and slope and to carry out research on the stress state, displacement and stability control of the reinforced slope.

## Figures and Tables

**Table 1:** Design parameter of slope.

Slope height (m)	Cohesive force kN/m^2^	Internal friction angle (°)	Gravity kN/m^2^	Limitation friction resistance kN/m^2^	Slope angle (°)
12	15	24	16.5	50	80

**Table 2:** Anchor arrangement for slope excavation stages.

Anchor serial number	The height from anchor top to slope (m)	Height of each stage of excavation slope (m)	Anchor layer number in each excavation stage
I	2.0	4.0	1
II	4.0	6.0	1*∼*2
III	6.0	8.0	1*∼*3
IV	8.0	10.0	1*∼*4
V	10.0	12.0	1*∼*5

**Table 3:** Anchor tension of slope excavation stages.

Excavation phase	Anchor tension of 1 layer (kN)	Anchor tension of 2 layer (kN)	Anchor tension of 3 layer (kN)	Anchor tension of 4 layer (kN)	Anchor tension of 5 layer (kN)	Security coefficient	Security coefficient
I	23.75					1.3136	1.6848
II	79.17	79.17				1.344	1.6456
III	71.25	142.50	106.88			1.3222	1.5981
IV	95.00	190.00	142.5	190		1.3424	1.45
V	79.17	158.33	237.5	158.33	197.92	1.3684	1.4209

**Table 4:** Different condition control parameters of sliding surface.

Working condition	Working phase	Sliding surface search variables			Slip plane center coordinates and radius			Security coefficient
		*X*	*Y*	*θ*	*x*_*c*_	*y*_*c*_	*R*	
A	I	2.8053	4.0	63.718	−11.575	11.102	16.039	1.3136
	II	3.458	6.0	90	−3.4764	6	6.9344	1.344
	III	5.6106	8.0	68.098	−14.328	16.016	21.49	1.3222
	IV	5.0633	10.0	90	−7.3434	10	12.407	1.3424
	V	7.8159	12.0	69.327	−23.373	23.769	33.335	1.3684

B	I	3.1053	4.0	61.633	−10.456	11.322	15.412	1.6848
	II	3.258	6.0	90	−3.8959	6	7.1539	1.6456
	III	5.4106	8.0	72.964	−10.349	12.829	16.483	1.5981
	IV	6.5633	10.0	73.361	−13.449	15.981	20.887	1.45
	V	7.2159	12.0	74.49	−18.013	19.001	26.182	1.4209
